# Notes on Some Cases from an Electrical Department

**Published:** 1904-06

**Authors:** George Parker

**Affiliations:** Physician to the Bristol General Hospital


### NOTES ON SOME CASES FROM AN ELECTRICAL DEPARTMENT.

BY

George Parker, M.A., M.D. Camb.,

*Physician to the Bristol General Hospital.*

---

In the last twelve months some two hundred patients were sent to this department at the General Hospital, comprising a great variety of cases. Many of these expected a "lightning cure," or were prevented from coming regularly, so that their attendance was short and useless. But a large number showed definite improvement, and some a complete recovery. Some were sent to me for diagnosis and then referred back to their physicians and surgeons, and many others I was asked to treat by my colleagues. There is a tendency to expect either marvellous cures, or to deny the efficacy of electrical methods altogether, whereas by patient work and by adapting the treatment to the individual case successful results, or at least steady improvement, will be obtained in a certain proportion of patients, though of course some failures are sure to be met with. The regeneration of a divided nerve or the growth of a wasted muscle is a very slow process at best, especially when the muscle has been left unused for years, or when the nerve which supplies it is still subject to the occasional influence of a poison such as we have in alcohol, diabetes, lead or rheumatism. Then in traumatic cases there are other difficulties, such as adhesions, habits of using other muscles or another limb, which have to be overcome, so that, though structural repair may be complete, you may have to wait a long while before the function is once more normal.

Our apparatus consists of a galvanic current led from cells to various stations; a Faradic current; and a secondary current derived from the sinusoidal street mains interrupted by a clock-work metronome and supplying a dozen arm and foot baths, and a full-length bath. The latter has also the galvanic current and a hot or cold electric douche. There is also an apparatus for high-frequency currents with an auto-condensation chair, and a Philips' hot-air bath giving a temperature of 300° from an Omega stove. Hot-air treatment is also given in bed in the wards by a double row of electric lights under a cradle, and electrical treatment from the sinusoidal current can be also given in bed through a transformer switched on to any wall plug.

**Duchenne's and Erb's Paralyses.**—I showed recently a little girl with almost complete recovery after prolonged treatment by electrical arm-baths and massage. Generally the prognosis is good if the injury has not been extreme. There is a difficulty in distinguishing these cases from infantile paralysis, in which recovery is slower and less complete. Warrington points out that there may be no loss of sensation even in these local nerve injuries, and pain cannot always be relied on in young children, and indeed it occasionally complicates infantile paralysis. If the lesion affects more muscles than those supplied by the fifth and sixth nerves, such as the triceps, I suppose we are justified in diagnosing infantile paralysis in doubtful cases. Harris and Low apparently prove that the lesion is confined to the fifth nerve only, and that in obstinate cases this nerve may be exposed, divided and sutured into the sixth or seventh. Tubby has succeeded in grafting a strip of the triceps into the biceps, and replacing the deltoid by portions of the pectoralis major and trapezius. I hope that my surgical colleagues may utilise some such procedure for intractable cases.

**Progressive Muscular Atrophy.**—I have rather discouraged the attendance of these patients as useless, but in one or two cases some arrest or improvement was shown. A patient of Dr. Neild's of the lower arm type quickly mended, the grasp of the left hand became 105, and that of the right 145. I have also notes of improvement in one or two women who attended, but a cure of the complaint is not to be expected.

In **Neuralgia** pure and simple the high-frequency currents seem at times to give immediate relief. I have personally experienced their value in severe faceache, and each application seems to give a longer interval of ease. The effleuve from the resonator is perhaps the best way of applying them.

In simple **Lumbago** rapid relief is obtained. I had several cases where good results followed a few hot baths through which the sinusoidal current was flowing, and there is probably no better cure for this painful condition than these baths. The great improvement in general health which patients show who have taken a course of these baths is very striking, and it seems to me that here in Clifton where baths with the sinusoidal or combined currents can be obtained at the Spa we are very behind-hand in making so little use of them in our daily practice. Gowers has broached an interesting theory of lumbago as due to compression of the muscle spindles, in which the afferent nerves start, by an inflammatory affection of the interstitial tissue, a fibrositis as he would call it. The final proof of this needs much pathological research, but I should like to direct attention to one form of the disease which he mentions, namely brachial myalgia, commencing with pain and tenderness in the insertion of the deltoid, and often mistaken for an affection of the joint. I have had some of these cases recently where the joint was quite free, but the arm was kept in a fixed position. The least movement sometimes causes intense pain unless the strain is taken off the affected muscle and its opponents.

**Diabetes.**—A few cases only were tried with high-frequency currents, and good results were noted in diminution of the sugar and gain of weight. Without claiming for the treatment the power of cure except in peculiarly favourable cases, it seems to be a valuable help by increasing the effect of diet and drugs.

R. B., a male, 65, attended as an out-patient for three years. The sugar was kept down by careful diet, opium and uranium nitrate; but from June to September it averaged 7 per cent., and his weight had fallen to about 7½ stones, though he was taking cod liver oil at the time. Without altering his other treatment, he received the high-frequency currents by the auto-condensation couch, and his fortnightly weight and percentage of sugar registered afterwards—

| | | | | | | | | | |
|---|---|---|---|---|---|---|---|---|---|
| Stones ... | 7-8 | 7-11 | 7-12 | 8-0½ | 8-4 | 8-7 | 8-7 | 8-6 | 8-8 |
| Sugar per cent. | 6½ | 5½ | 6½ | 6 | 4½ | 6 | 4 | 4½ | 4½ |

or a gain in weight of a stone, while the sugar averaged 5.3 per cent.

E. G., a female, 58, showed lung symptoms with much blood-stained expectoration besides the glycosuria. There were no bacilli, however, and on restricted diet and rest in bed the lung symptoms cleared up and she gained in weight, but until the blood ceased I did not venture to use the high-frequency currents. She did very well, and soon left the hospital with only a trace of sugar, but her gain of five pounds in three weeks cannot be put down to the use of the currents, as she was putting on flesh rapidly before.

In the same way it is difficult to estimate the value of the currents in a case of diabetes insipidus, for the treatment was only employed after an improvement under drugs was started. However, the patient gained two stones in weight, and the amount of urine fell from 132 ounces daily to 86. He went back to work, but comes to see me from time to time.

**Functional Cases.**—Some of these were most curious. A man with marvellous clonic spasm of the eyelids was treated with the high frequency effleuve and steadily improved, leaving well and fit for work; while a girl of twelve with left hemiplegia and anæsthesia complete in the arm can be cured daily by the same treatment, but comes back regularly as bad as ever. I am hopeless about her unless she can be sent away from home for some months and isolated from her friends. Another girl, in whose case there seems some underlying Raynaud's disease, had both hands red and cyanosed. This condition had previously appeared in the right hand every October for some years and passed away again, but when she came to me the right hand was firmly clenched, and the arm and hand were much swollen and pitted on pressure. On the dorsal surface were several blebs. The sense of touch and of heat were normal, that of pain indistinct at first but afterwards normal. The Faradic and galvanic reactions were normal, but the spasm continued for months, and was only slightly relieved by treatment. Our baths, I should say, were not then in full working order. After a time the entire group of symptoms passed away, and I have lately seen her apparently quite well, two fingers only being a little stiff after their long continued contraction. The following case in which rupture of the brachial plexus was negatived by the electrical reactions was probably functional.

H. T., 29, a telephone fitter, on trying to turn round in a subway, on February 24th, wrenched his right arm backwards and downwards. He was sent to me by Dr. Symes about a month after his accident complaining of complete loss of the power of movement in the right arm and hand. On April 6th there was no definite wasting of the muscles, but a slight protrusion of the posterior border of the scapula, possibly due to the position in which he stood. The Faradic current, when one electrode was placed over the plexus above the clavicle and the other on the back, gave a reaction in the muscles of the injured arm equal to that obtained on the sound side. The Faradic reactions in the deltoid, biceps, triceps, extensors and flexors of the hand and fingers, and in the thenar muscles were all normal. The only galvanic change noted was that the minimal current required to give a reaction in the lower part of the limb was rather greater than on the sound side. Thus there was no reaction of degeneration six weeks after the injury, and the conductivity of the cord was unimpaired. The elbow appeared slightly abducted and the wrist a little flexed. Sensation, according to the patient's account, was lost below a horizontal line round the arm touching the anterior and posterior folds of the axilla. Passive movements were free, and no dislocation or injury to the joints of the arm was present. The patient was given a favourable prognosis, but disappeared for twelve months, when he was found to have regained complete use of the arm.

I am in doubt whether the next case was functional or an occupation palsy.

E. M., 48, a carpenter, lost sensation in the index finger of the left hand, so that he had to hold nails between the thumb and second finger. Eighteen months later this hand became suddenly clenched while he was at work and remained in that condition. There was now a loss of sensation to pain, heat and touch up to a horizontal line round the elbow, but rather higher on the outer and posterior sides. The electrical reactions were fairly normal. After a fortnight's treatment by arm baths the reactions were still normal, and there was some return of movement, no pain, nor any wasting. The patient felt much better, the grasp of the left hand equalled 40, of the right 110. Tactile sensation was good down to a horizontal line two inches below the elbow.

This circular line of anæsthesia, corresponding to no nerve distribution, has been taken for a sign of hysteria, but I think it may occur otherwise in rare instances. We noticed it in a case of birth palsy and after the accidental division of a nerve during an operation, the patient being a merry, matter-of-fact lad, not very sensitive to pain.

**Facial Paralysis.**—Here in simple cases, if there is no reaction of degeneration, the results are uniformly good. The positive pole with not more than five milliampères of current is moved up and down over the pes anserinus. In crutch paralysis the arm bath is often sufficient, or the constant current may be applied transversely where the musculo-spiral crosses the outside of the arm. One remarkable case of paralysis of the serratus magnus after an injury gave the typical winged scapula when the arm was raised in front of the patient, and recovered power very slowly under local treatment.

**Birth Palsy** (Little's disease).—Few things need more care and patience in treatment than this, almost every muscle wants separate attention. The spastic condition of the limbs may depend on one or two muscles, the rest of the flexors perhaps, and all the extensors may be wasted, and it is very difficult to pick out the wasted ones and exercise them.

O. O., a boy of 12, came to me unable to sit up alone in a chair, though he crawled about on his knees and the back of his wrists. There was a spastic condition of all four limbs, but the left arm was less affected than the rest, and as he had good intelligence he had learnt to write a little with the left hand. After prolonged treatment he can now stand alone and walk with crutches, and is steadily gaining power. The contractions have been largely overcome by splints, and by exercising the weak muscles from the sinusoidal current. Well wetted electrodes are tied over the selected muscles, and as the street current is reduced to about ten volts or less and cut off every two seconds, the muscles can be worked as in ordinary exercise as long as is desirable. Massage too has effected a great improvement, and as the spastic condition passes off rhythmic exercises are most necessary. Tenotomy may be needed in some cases, but much may be done without it by splinting and by improving individual muscles.

**Lead Poisoning.**—Since even the poultry in some parts of the Mendips are said to be killed by the lead they pick up, we cannot be surprised at the number of cases of lead paralysis which we meet with. Some of them are very slow to mend under any treatment. How is it that in a certain number we find reaction of degeneration in some extensor muscles, the rest, like the flexors reacting normally? I saw a patient last week whose grasp was only one quarter of the strength it should be. The electrical reaction of the flexors was normal, while every muscle on the back of the arm showed R. D. except the extensor ossis metacarpi, and the extensor carpi ulnaris. Each finger, too, differed from the rest in the amount of the drop, illustrating the extreme variations in the action of the poison on the individual nerve-muscle. I have obtained improvement in the dropped wrist in some cases by fixing the hand in a hyper-extended position on a splint "to take up the slack" of the over-stretched extensors, but the question is, How long can this splint be kept on without weakening the whole arm? Probably the best plan is to keep it continuously applied and stimulate the muscles daily at the same time until the drop has gone. Then constant exercise, such as carrying a bucket and squeezing a ball, should be added to the electrical treatment.

**Rheumatoid Arthritis.**—Here the results were most variable. Some recovered more or less completely, others showed no effect except that nearly all got more or less relief from pain. Some of the septic cases showed extraordinary improvement after a course of electric full-length baths, whilst the results from hot-air baths were less satisfactory, though in old quiescent cases with much anchylosis hot air may be valuable with or without a previous breaking down of adhesions, provided that some treatment is also applied to the wasted muscles, such as arm baths or massage. A difficult type to treat is that where deep mental depression exists. These patients are very sensitive to pain, and cannot be induced to keep up any movements. If a joint is loosened to-day they keep it motionless until it is again fixed, however free passive movements are at first. High feeding, tonics, baths for the whole body with the combined current and massage seem to be the most effective treatment even for these.

**Joint Cases.**—For adhesions after the results of an injury have quieted down, hot air gives painless and steady improvement, though in some cases it may save time to break down the adhesions under ether and then complete the cure by hot air. In the electrical treatment of joints the constant current only is of use, and preferably this should be combined with cataphoresis. A nickel electrode with a pad soaked in iodide of potash or salicylate of soda is used as the positive pole, and a current of twenty milliamperes is sent through the joint. The poles must be reversed every five minutes for a short time to avoid certain results of polarisation. The very painful shoulders which follow an injury and subsequent attacks by some rheumatic or rheumatoid affection are thus relieved. I have had patients recently who under hot-air treatment followed by cataphoresis lost the pain and regained movement in a short time.

Mrs. C. came into the ward with both hip joints anchylosed, the right completely. The legs were usually crossed as in spastic paraplegia, but the reflexes were normal. She complained of paroxysmal pain down the thighs. How she contrived to sit or move at all was puzzling. I gave her hot-air baths in bed and massage and passive movements daily, under which the bones grated like a rusty hinge. The pains disappeared, the adhesions rapidly gave way, and in two or three weeks she walked about with a stick.

In conclusion, the curious results of the loss of the pain sense in a case of syringomyelia may be noticed. The patient was treated by arm baths for rigidity of the muscles of the hands, though there is no reason to expect there would be any effect on the course of the disease itself.

S. L., 30, a crane driver, suffered five years ago from spontaneous fracture of the right ulna, but feeling no pain he went on working for hours to the damage of his tissues. Three years ago the left arm had a similar accident, and his fingers kept getting crushed in cogwheels without his knowledge. He used to light his pipe, like his mates, by picking up quickly a hot cinder with finger and thumb, but found he burnt holes in them as he did not know when to drop it. In one of these many accidents a little finger became poisoned and was left contracted. Seven weeks ago both hands became swollen, red, and dry, and all the fingers became fixed. Then crops of vesicles appeared on the region of the right radial nerve, and a few on that of the left. The feet were never affected, and there was no spastic state of the legs, the knee jerks being normal. The heat sense was absent on both surfaces of the hands and arms, defective over shoulders and front of thorax and down nearly to the umbilicus. The muscular sense was normal. There was some tactile anæsthesia when first examined in the area of both median nerves, wasting of the first interosseous and thenar muscles, and trophic changes in right elbow.

After arm baths for fourteen days he regained fair movements in the fingers. Otherwise he remains the same, and perpetrates fresh fractures and wounds quite cheerfully.

